# Exploring the impact of gamified elements on motivation-related variables in physical education

**DOI:** 10.1371/journal.pone.0358950

**Published:** 2026-09-23

**Authors:** Mario Tudela-Petit, Adrià Marco-Ahulló, Hugo Centelles-Lodeiro, Xavier García-Massó

**Affiliations:** 1 Department of Teaching Physical Education, Arts and Music, Universitat de València, València, Spain; 2 Department of Personality Psychology, Treatment and Methodology, Universidad Católica de Valencia, València, Spain; Farhangian Teacher Education University: Farhangian University, IRAN, ISLAMIC REPUBLIC OF

## Abstract

Gamification in physical education is an active methodology that fosters student engagement. However, few studies have examined its influences based on the types of elements in gamification. This study analyzed and compared the effects of these factors on motivation, future physical activity intention and basic psychological needs. The intervention was conducted with secondary school students in Spain during 8 physical education sessions in different schools. The 209 participants were divided into 3 groups: a control group (CG), a group where intrinsic gamification elements predominated (InMG), and another where extrinsic elements predominated (ExMG). Participants completed the questionnaires before and after the intervention. The results revealed a significant time × group interaction effect for perceived competence (p = .023) and future physical activity intention (p = .02). Although these interactions did not derive from statistically significant post-hoc pairwise comparisons, descriptive trends revealed a tendency toward an increase in perceived competence for both the ExMG and InMG, and a specific upward tendency for future physical activity intention only within the InMG. No significant effects were observed for the remaining motivational regulations, which remained stable across all conditions. In conclusion, the findings delineate the specific and nuanced impacts of the two evaluated gamification profiles. While both the InMG and ExMG frameworks were associated with a descriptive tendency toward a slight improvement in students’ perceived competence compared to the CG, the InMG profile suggested a possible descriptive advantage in fostering intentions for future physical practice that requires further confirmation.

## Introduction

Despite the well-documented physical, psychological, and social benefits of regular physical activity (PA), adolescents often experience a progressive decline in motivation and engagement during secondary school in PE classes [1–3]. To address this disengagement, frequently driven by traditional, repetitive, or overly competitive teaching environments [4,5], pedagogical approaches can benefit from fostering learning environments designed to support students’ basic psychological needs (BPN) (autonomy, competence, and relatedness), as proposed by Self-Determination Theory (SDT) [6]. According to this framework, satisfying these three needs is essential to foster self-determined forms of motivation, which in turn lead to deeper engagement and positive behavioral outcomes. Building on the work of Zourmpakis et al. [7], who highlight the critical role of carefully designed instructional environments in shaping student motivation, gamification has emerged as a prominent active methodology to achieve this goal [8–12].

However, although this approach is widely identified as a positive tool, authors such as Zhang and Yu [13] emphasize in their meta-analysis that its motivational effects depend on the underlying pedagogical design. For instance, interventions predominantly based on points, badges, and leaderboards (PBL) tend to increase extrinsic motivation but show inconsistent or limited effects on intrinsic motivation [13]. In contrast, gamified designs that incorporate elements such as missions, narratives, and collaboration appear to better support students’ BPNs [14]. Reinforcing this idea, Huang [15] found that collaborative and voluntary tasks amplified engagement more effectively than competitive leaderboards.

This divergence can be explained through SDT’s Cognitive Evaluation Theory [16]. External elements like PBL are often interpreted as normative and controlling, especially when used competitively, which can thwart the basic need for autonomy and reduce intrinsic motivation [17]. Conversely, elements such as narratives, missions, voluntary challenges, and social collaboration tend to be experienced as more autonomy-supportive and meaningful, thereby contributing to the satisfaction of BPN and fostering intrinsic motivation [18–20]. Thus, it appears that gamified features possess the potential to satisfy basic psychological needs such as autonomy and competence, particularly when their elements are deliberately designed to be informational rather than controlling [21,22]. Therefore, it is not merely the inclusion of game elements that matters, but how these elements are designed and implemented, and how they are interpreted by students in the learning environment [15].

Given the differentiated effects that gamified elements can have on students’ motivation depending on their design and interpretation, it is crucial to follow a structured and theory-informed approach to gamification design in educational settings. Specifically, there is a need to select elements that are more likely to support the satisfaction of the BPN and thereby promote intrinsic motivation. One useful tool for this purpose is the Octalysis Framework, developed by Chou [23], which provides a systematic way of analyzing and designing gamified experiences based on eight core drives of human motivation. These drives (epic meaning, accomplishment, empowerment, social influence, scarcity, unpredictability, avoidance and ownership), can be mapped onto the SDT framework to identify which elements are more aligned with intrinsic or extrinsic motivation. For example, narrative elements and voluntary challenges can foster creativity and a sense of autonomy, feedback mechanisms enhance students’ competence, and social collaboration and mentorship promote social influence and relatedness. By using frameworks like Octalysis, educators can move beyond superficial or reward-based gamification and create more meaningful and psychologically supportive learning environments, especially in contexts like PE, where motivation is central to fostering lasting behavioral change.

Despite these theoretical foundations, a significant research gap remains. Comparative studies that empirically test and isolate different gamification profiles (e.g., intrinsic-oriented vs. extrinsic-oriented) within the same ecological educational context are scarce, particularly in PE. Therefore, it is necessary to advance beyond treating gamification as a uniform intervention, and instead investigate how specific configurations of game elements influence the longitudinal development of motivation and behavioral intentions.

Consequently, the aim of this study was to examine the differential effects of two gamified interventions [one based on extrinsic elements (e.g., PBL) and one based on intrinsic elements (e.g., narratives, missions, unlockables, social interaction)] on students’ motivation, BPN satisfaction, and future intentions to engage in PA.

We hypothesized that students’ psychological and motivational variables would show differential trajectories across the assessment periods depending on the gamification profile. Specifically, the intervention based on intrinsic elements was expected to lead to positive changes in autonomy, competence, and relatedness satisfaction, resulting in higher levels of intrinsic motivation and stronger future intentions to engage in PA, and lower levels of amotivation compared to both the control and extrinsic conditions. In contrast, the extrinsic-based intervention was anticipated to result in an increase in extrinsic regulation, showing weaker effects on intrinsic variables, while amotivation was expected to remain stable or follow an increasing trajectory in both the extrinsic intervention and control group.

## Materials and methods

### Participants

The initial sample consisted of 217 students, recruited between 13 and 29 March 2024 from four different secondary schools in the province of Valencia (Spain). Only participants who met the following inclusion criteria were included in the final analysis: (a) attended at least 75% of the PE classes; (b) their legal guardians signed the informed consent document; and (c) completed the questionnaires both in the pretest and posttest. A total of 8 students were excluded for not meeting the attendance criterion, resulting in a final analytical sample of 209 students (106 boys: 50.7%, and 103 girls: 49.3%) with an average age of 15.5 years (SD = 0.95)

A priori power analysis was conducted using G*Power 3.1.9.7 (University of Düsseldorf, Düsseldorf, Germany) to determine the required sample size for an ANCOVA with three groups and one covariate. We set the significance level at α = .05, statistical power at 1 − β = .90, and specified an effect size of f = 0.27, which was derived from a previous study examining the effects of gamification on students’ intrinsic motivation [14]. The numerator degrees of freedom were set to df = 2 (based on the three-group design), and one covariate was included. The analysis indicated that a minimum sample size of 177 participants would be required to achieve adequate statistical power. Given potential attrition and to ensure robustness of the results, we recruited a total of 209 participants, exceeding the required minimum sample size. While the primary analytical strategy was subsequently expanded during peer review to Linear Mixed Models and RM-ANCOVAs to account for complex random structures and baseline covariates, this initial power calculation served exclusively as a pre-study planning estimate to ensure adequate sample size before data collection. Additionally, the study was approved by the Ethics Committee of the University of Valencia (2023-MAGPED-3167180).

### Study design and procedure

This research was designed and conducted during the 2023–2024 academic year, in the PE subject, with students in years 10–12, following the Spanish secondary education curriculum. A total of 11 entire classes from these academic years participated. The study design was quasi-experimental. Because students were already clustered within pre-existing educational groups, individual randomization was not possible. Therefore, the intact class served as the exact unit of assignment. These intact classes were randomly assigned to the different intervention conditions, allowing for maximum control considering the nature of the academic context [24].

Different aspects were considered to ensure the proper functioning of the research. First, pretests and posttests were administered to all groups. Both in the pretest and posttest, participants completed questionnaires on motivation in PE, satisfaction of BPN, and future intention to engage in PA. It is also relevant to mention that the main instructor was the actual teacher from the educational institution. While using the regular teacher helps maintain ecological validity and avoids the novelty effect of an external instructor, teacher-related variability across the 11 classes remains a potential confounding factor. To monitor and mitigate this variability, a single member of the research team was present to oversee all intervention sessions across the participating schools, ensuring the pedagogical and gamified elements were implemented consistently.

### Educational programme

The intervention comprised eight 55-minute sessions conducted over four consecutive weeks. A total of 11 secondary school classes (from four different schools) participated in the study. These classes were allocated via stratified random assignment at the class-level (ensuring that classes from the same school were distributed across different conditions) into three conditions: a control group (CG), an experimental group with intrinsic gamification (InMG), and another with extrinsic gamification (ExMG).

The pedagogical layer of the intervention applied to all groups was framed within the official PE curriculum of the Valencian Community (Decree 107/2022, DOGV), which implements the LOMLOE for compulsory secondary education. Specifically, the selected contents focused on throwing techniques and their application to various individual and team sports, which are part of the basic knowledge (technical foundations of sport and tactical foundations of sport) associated with the second specific competence of the subject.

The intervention addressed learning situations related to object control skills, particularly those involving accuracy, power, and adaptation of throwing techniques to different tactical and physical demands. These skills contribute directly to criterion of evaluation 1.2.

In line with the curriculum's competency-based approach, students were not only expected to reproduce throwing techniques, but to adapt them to solve practical challenges across a range of game formats and PA settings. These included handball-based games, frisbee activities, and track-and-field-inspired throwing circuits. The design of the intervention thus reflects both conceptual and procedural content, in accordance with the learning situations and expected learning outcomes set out for the 3rd and 4th years of secondary school in the Valencian curriculum.

Nevertheless, the gamification layer was different between the groups. The CG followed the intervention without the integration of any gamified components or digital support. This group served as a baseline for comparing the effects of gamification on students’ motivation, BPN and future intention to be physically active. The ExMG group participated in the same PAs but through a gamified environment oriented toward extrinsic motivation. Utilizing the MyClassGame platform (configured in this condition to focus on an extrinsic profile) game elements such as experience points, health points, exchangeable coins, and virtual collectibles were implemented. Students accumulated rewards based on their participation and performance, which were reflected in leaderboards updated weekly. These elements emphasized measurable progress, competition, and tangible outcomes, aligning with Octalysis core drives 2 (accomplishment), 4 (ownership), and 6 (scarcity). The primary goal of this design was to stimulate external regulation by reinforcing behaviors through incentives and recognition.

**Fig 1:**
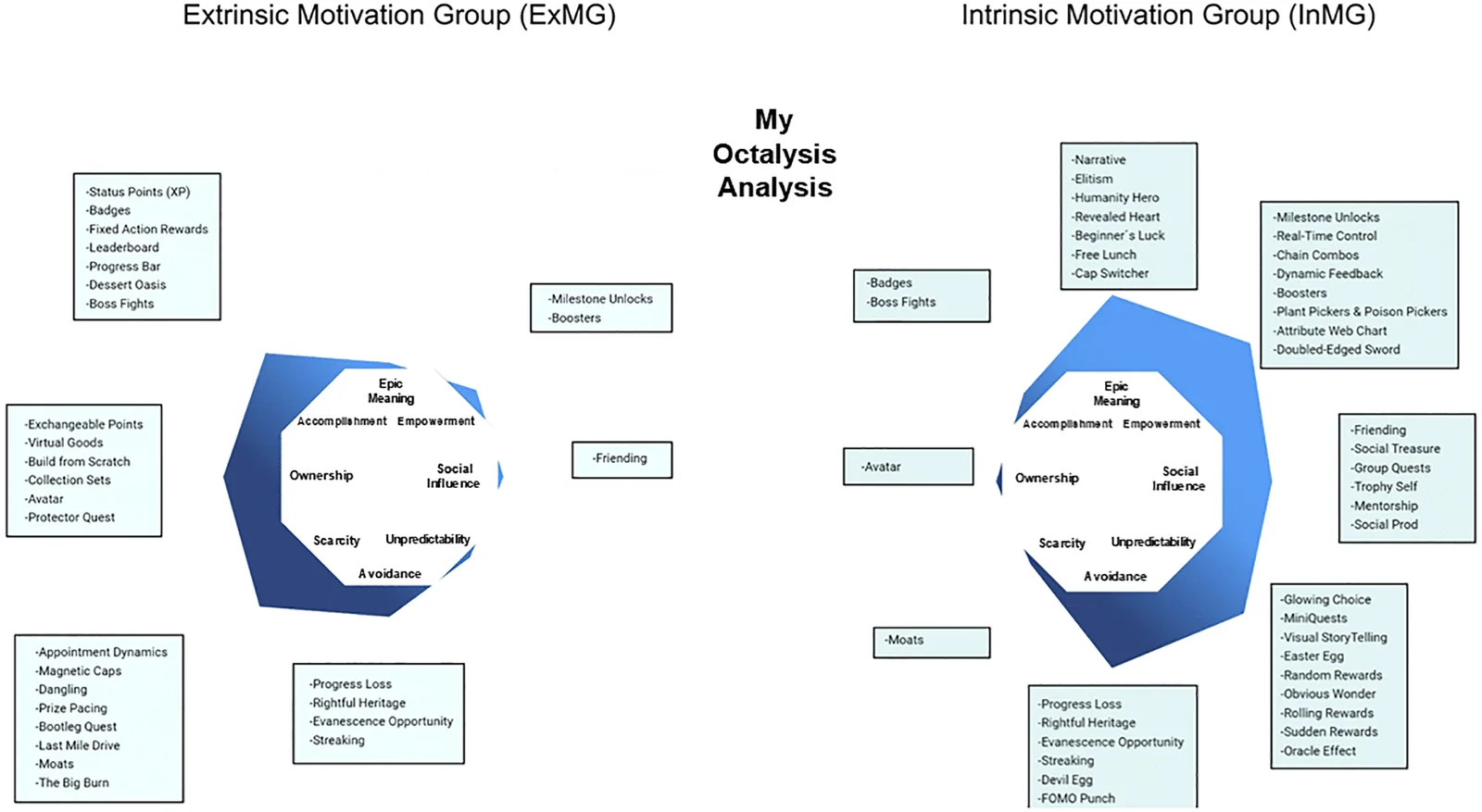
Differences between intrinsic and extrinsic motivation groups in terms of game elements.

Conversely, the InMG group experienced the same curricular sequence within a narrative-driven gamified framework designed to enhance intrinsic motivation, utilizing the same MyClassGame platform but configured to support intrinsic mechanics. Students took on the role of heroes in a fictional storyline, unlocking chapters and facing “boss fights” as they progressed through cooperative physical missions. The intervention emphasized autonomy-supportive mechanics such as voluntary challenges, dynamic feedback, and student-driven decision-making, along with social elements like mentorship, collaborative quests, and friending. These elements were mapped to Octalysis core drives 1 (meaning), 3 (empowerment), and 5 (social influence) and aimed to satisfy the BPN of autonomy, competence, and relatedness, in accordance with SDT [6]. Fig 1 highlights the most notable elements within each of the experimental groups. While the experimental conditions were designed to isolate intrinsic and extrinsic orientations, certain structural elements (e.g., badges) were present in both groups to provide necessary scaffolding; however, their implementation fundamentally differed (narrative-driven vs. normative evaluation). To provide a clear distinction of these dynamics, Table 1 summarizes the unique and shared game elements applied in each condition. Furthermore, the complete list of Octalysis Framework elements used in the two gamified groups and their description are provided in Supplementary Material (S1 File).

**Table 1:** Overview of gamified elements across the experimental conditions.

Element type	Intrinsic Gamification Group (InMG)	Extrinsic Gamification Group (ExMG)
*Unique elements*	Narrative, Elitism, Humanity Hero, Revealed Heart, Beginner’s Luck, Free Lunch, Cap Switcher, Real-Time Control, Chain Combos, Dynamic Feedback, Plant Pickers & Poison Pickers, Attribute Web Chart, Double-Edged Sword, Social Treasure, Group Quests, Trophy Self, Mentorship, Social Proud, Glowing Choice, Mini Quests, Visual Story Telling, Easter Egg, Random Rewards, Obvious Wonder, Rolling Rewards, Sudden Rewards, Oracle Effect, Devil Egg, FOMO punch	Status Points (XP), Fixed Action Rewards, Leaderboard, Progress Bar, Desert Oasis, Exchangeable Points, Virtual Goods, Build from Scratch, Collection Sets, Protector Quest, Appointment Dynamics, Magnetic Caps, Dangling, Prize Pacing, Bootleg Quest, Last Mile Drive, Big Burn
*Shared elements*	Badges, Boss Fights, Milestone unlocks, Boosters, Avatar, Friending, Moats, Progress Loss, Rightful Heritage, Evanescence Opportunity, Streaking	
*Implementation of shared elements*	Used as narrative milestones to unlock chapters and foster a sense of journey.	Used as normative evaluations to rank performance and accumulate status.

To monitor the intervention process, the same researcher oversaw all sessions in both gamified groups, and each class teacher was responsible for routine group management. This tracking was performed continuously by verifying student session attendance and checking the last access records from MyClassGame to ensure that participants successfully accessed the specific digital components assigned to their respective experimental conditions. However, it must be explicitly noted that a formal, systematic fidelity assessment was not conducted. Consequently, these indicators primarily confirm students’ exposure to the digital components and general attendance, rather than the precise pedagogical fidelity with which the intervention was delivered.

### Measuring instruments

The data were collected from 3 different measurement instruments at two different times: before (24–48 hours before) and after (24–48 hours after) the intervention. Each of the instruments used is described below.

#### Physical activity psychological need satisfaction questionnaire.

The instrument validated by Sebire et al. [25] was used to quantify the perception of satisfaction of BPN in students in the context of PE classes. This instrument is composed of 16 items: 6 items that express autonomy, 5 items for competence and 5 items for relatedness. Confirmatory factor analysis indicated acceptable adjustment of the data (χ2/df = 2.58; RMSEA = 0.052; SRMR = 0.04; CFI = .951) and good internal consistency of autonomy (α = .72), competence (α = .82) and relatedness (α  = .81) [25]. The data collected in the present study showed Cronbach's alpha values of .72, .84 and .93 for the autonomy, competence and relatedness variables, respectively.

#### Future physical activity intention.

This instrument is based on the work of Chatzisarantis et al. [26] and translated by Castillo et al. [27], in which participants respond to 3 items. The main objective is to assess the future intention to engage in physical-sporting activities. This instrument has shown acceptable reliability in the context of PE subjects in adolescents [26], and our data showed a Cronbach's alpha value of .94.

#### Perceived Locus of Causality Scale in PE (PLOC-R).

This instrument was used to assess different motivations in the context of PE, based on the SDT framework [28]. It consists of 20 items and encompasses 5 different dimensions of motivation: intrinsic motivation, identified regulation, introjected regulation, external regulation, and amotivation. No questionnaire items are reproduced in this article. A sample of 1535 students aged 12–17 years was used to validate the instrument. The results showed that it is a tool with adequate fit indices (χ2/df = 6.64; RMSEA = 0.06; SRMR = 0.05; CFI = .90; TLI = .88; IFI = .90). The internal consistency (Cronbach's alpha) values obtained in the present study were as follows: intrinsic motivation, α = .86; identified regulation, α = .83, introjected regulation, α = .70; external regulation, α = .71 and amotivation α = .83. These dimensions represent distinct, yet theoretically related, forms of regulation along the motivational continuum, ranging from the most autonomous motivation to the most controlled forms of regulation, including the absence of self-determined motivation. In the present study, these subscales were analyzed separately to examine whether different gamification profiles were associated with specific shifts along the motivational continuum. This distinction proved particularly relevant to the research aims, as the study sought to determine whether intrinsically oriented and extrinsically oriented gamified environments were associated with distinct patterns of motivational regulation, rather than a generalized change in motivation.

### Statistical analysis

Statistical analyses were conducted using JASP software (version 0.95.4; JASP Team, 2025). Given the quasi-experimental design of the study, we first performed a one-way ANOVA (factor: group; levels: control, InMG, and ExMG) to examine potential baseline differences in the dependent variables (i.e., BPN, motivational regulation, and future intention to engage in PA). As significant group effects were detected for some baseline measures (see Results), the primary intervention effects were evaluated using Linear Mixed Models (LMM). For the dependent variables, time (pre-test vs. post-test), group (control, ExMG and InMG), and their interaction (time × group) were specified as fixed effects. Baseline differences highlighted in Table 2 were formally controlled for by introducing the corresponding pre-test scores as fixed-effect covariates.

**Table 2:** Baseline data of motivation-related variables.

	Baseline		
	*Control*	*ExMG*	*InMG*
**Autonomy**	2.71 (.79)	2.97 (.59)	3.37 (.62)* †
**Relatedness**	3.64 (.99)	3.88 (.84)	3.77 (1.05)
**Competence**	3.86 (.85)	3.81 (.69)	3.85 (.81)
**Future PA intention**	4.20 (.91)	4.29 (.97)	4.17 (.93)
**Intrinsic Motivation**	5.00 (1.50)	5.21 (1.31)	5.17 (1.50)
**Identified Regulation**	5.23 (1.46)	5.03 (1.44)	5.20 (1.23)
**Introjected Regulation**	4.48 (1.33)	4.05 (1.30)	4.44 (1.39)
**External Regulation**	4.48 (1.35)	3.86 (1.42)*	4.13 (1.48)
**Amotivation**	3.14 (1.65)	3.04 (1.80)	3.06 (1.59)

**Note.** Data are expressed as mean (SD). * Indicates significant differences regarding the control group (p < .05). † Indicates significant differences regarding the ExMG (p < .05). InMG = intrinsic motivation group; ExMG = extrinsic motivation group.

Initially, both classroom membership (cluster level) and participant ID were specified as random intercepts to account for the nested and repeated-measures structure of the data. When classroom-level variance was negligible (ICC < .05), the cluster-level random intercept was removed to prevent overparameterization and singular fit matrices; ultimately, the classroom-level random intercept was retained exclusively for Perceived Competence and Relatedness. Importantly, the participant-level random intercept (ID) was systematically retained across all Linear Mixed Models to explicitly account for within-person dependence and protect standard errors from underestimation. For variables where matrix convergence issues persisted due to perfect baseline multicollinearity between the random intercept and its corresponding fixed covariate (specifically Autonomy and External Regulation), and given that classroom-level and/or ID-level intraclass correlation was negligible (<0.05) a Repeated Measures ANCOVA (RM-ANCOVA) was implemented as a mathematically robust alternative to maintain strict baseline control without overparameterization. In these models, the within-subject factor was time (pretest vs. posttest), the between-subject factor was group (control, ExMG and InMG), and potential baseline bias was controlled for by cross-introducing other relevant baseline measures as fixed covariates (i.e., introducing baseline Autonomy as a covariate in the External Regulation model, and vice versa), thereby avoiding mathematical redundancy while maintaining strict baseline adjustment. Statistical assumptions were systematically verified for all analytical frameworks. Prior to conducting the analysis, assumptions were checked. For Linear Mixed Models (LMM; Competence, Relatedness, Future PA Intention and motivational regulation), conditional residual normality was confirmed via Q-Q plot inspection across conditions, homoscedasticity was verified through residual-versus-fitted plots, and multicollinearity was ruled out (Variance Inflation Factors < 3.0). Sphericity was neither assumed nor required for LMMs given their flexible variance-covariance structure. For the Repeated Measures ANCOVA (autonomy and External Regulation), distributional normality of residuals was similarly verified via Q-Q plots, sphericity was held by design (k = 2) and homogeneity of variances was confirmed via Levene’s test. For the linear mixed models and RM-ANCOVAs, partial eta-squared (η^2^_p_) was reported as the index of effect size. Follow-up pairwise comparisons for the significant interaction effects were calculated via planned linear contrasts using the models’ estimated marginal means and pooled standard errors, applying Holm’s correction for multiple testing. Statistical significance was set at p < .05 for all analyses.

## Results

### Baseline differences

The main results of the three groups in the pretest (CG, ExMG, and InMG) are presented in Table 2, including the significant differences. There was a significant difference between the groups in terms of the autonomy variable, with the InMG scoring higher than the CG (p < .001) and the ExMG (p = .002). Finally, the ExMG scored significantly lower than the CG in terms of external regulation (p = .028).

### Basic psychological needs

**Fig 2:**
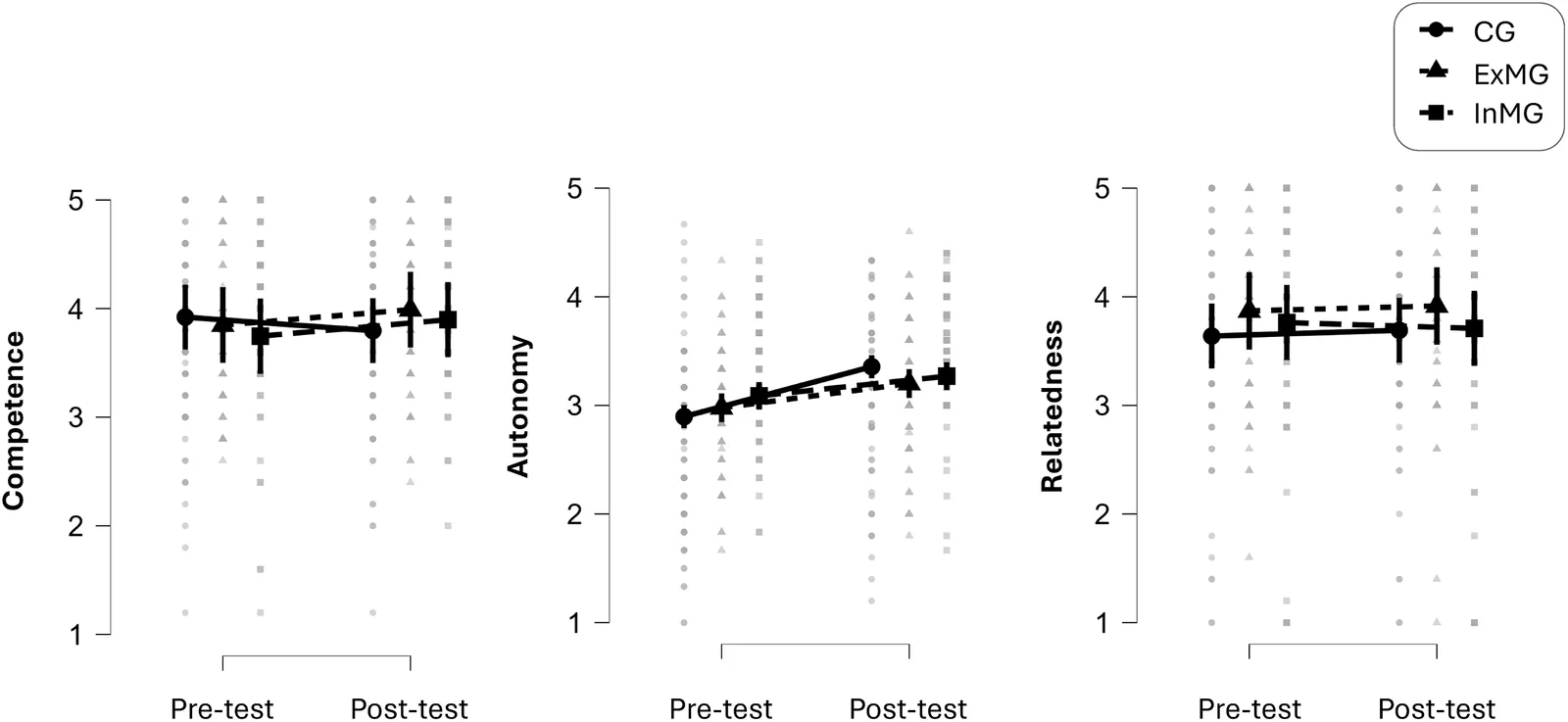
Jitter plot of basic psychological needs.

The repeated measures ANCOVA showed that the interaction effect between group and time was not significant on the perception of autonomy (F2,204 = 2.47; p = .11; η^2^_p_ = .02). The linear mixed models showed that there was no significant interaction effect of group and time on the perception of relatedness (F2;205 = .34; p = .71; η^2^_p_ = .003). However, a significant interaction effect of group and time was found on the perceived competence variable (F2;206 = 3.84; p = .023; η^2^_p_ = .036). Pairwise comparisons revealed no significant differences between groups at either pre-test or post-test, nor were any significant within-group changes observed from pre-test to post-test. However, a descriptive trend (Fig 2) indicated that both experimental groups improved their perception of competence between the pre-test and post-test, whereas the control group experienced a decline.

### Future physical activity intention

**Fig 3:**
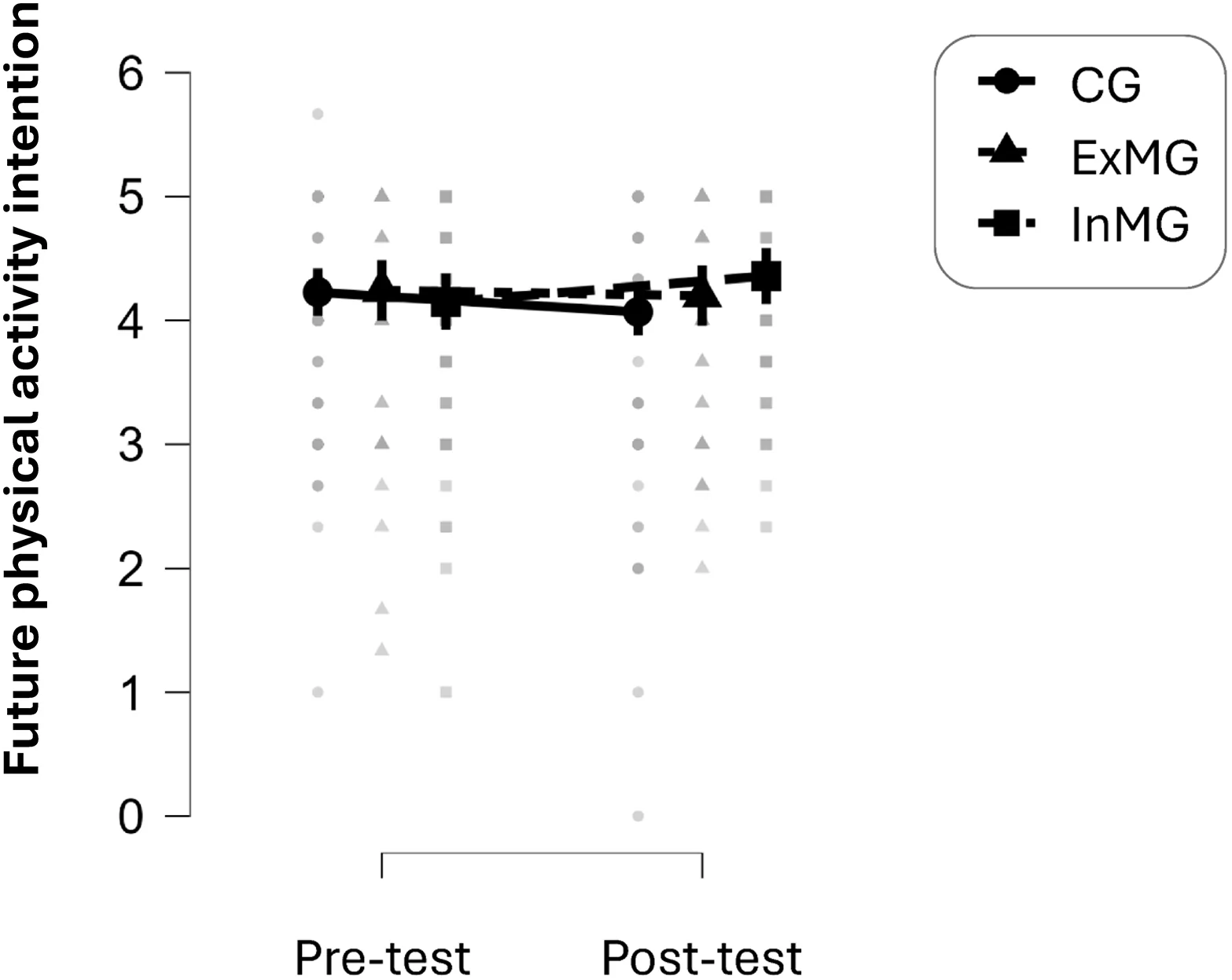
Jitter plot of future PA intention.

The linear mixed model showed a significant interaction effect of group and time on the future PA intention (F2;203.45 = 3.91; p = .02; η^2^_p_ = .037). Although no significant differences were observed between groups at either pre-test or post-test, and no group showed significant within-group changes over time, Fig 3 illustrates a descriptive divergence: while the InMG group increased their future PA intention, the other two groups displayed a downward trend.

### Motivation towards physical education

No effect of group * time was found on any of the variables related to PE motivation (see Table 3). The descriptive statistics associated with these variables are shown in Table 4.

**Table 3:** Findings from LMM and RM-ANCOVA (for external regulation) analysis of motivation towards physical education variables.

Variable	F_2,205_	p-value	h^2^_p_
**Intrinsic Motivation**	.17	.85	.002
**Identified Regulation**	.2	.82	.002
**Introjected Regulation**	1.7	.19	.016
**External Regulation**	.7	.49	.007
**Amotivation**	0.44	.64	.004

**Table 4:** Descriptive statistics (mean and SD) of motivation towards physical education.

	Time	CG	ExMG	InMG
**Intrinsic Motivation**	Pre-test	5.27 (1,22)	5.22 (1.19)	4.78 (1.25)
	Post-test	5.38 (1.22)	5.22 (1.19)	4.81 (1.25)
**Identified Regulation**	Pre-test	5.47 (1.21)	5.03 (1.19)	4.85 (1.24)
	Post-test	5.49 (1.21)	5.17 (1.19)	4.92 (1.24)
**Introjected Regulation**	Pre-test	4.55 (1.24)	4.18 (1.22)	4.25 (1.27)
	Post-test	4.59 (1.24)	4.47 (1.22)	4.15 (1.27)
**External Regulation**	Pre-test	4.28 (0.92)	4.13 (0.92)	4.19 (0.93)
	Post-test	4.13 (0.92)	4.25 (0.92)	4.11 (0.93)
**Amotivation**	Pre-test	2.88 (1.53)	3.17 (1.51)	3.31 (1.55)
	Post-test	3.08 (1.53)	3.26 (1.51)	3.26 (1.55)

Finally, a comprehensive summary detailing the Estimated Marginal Means, standard deviation, *p*-values, and effect sizes for all post-hoc pairwise comparisons across all dependent variables is available in Supplementary Material (S2 File).

## Discussion

The main objective of the present study was to analyze the differential effect between two types of gamification, one focusing on more intrinsic motivation-oriented elements and the other characterized by extrinsic motivation-oriented components (with PBL-type elements), and to compare them with a group that did not experience either gamification, observing the effect produced in terms of BPN, future PA intention, and motivation towards PE on this subject.

The results showed a significant time × group interaction effect for perceived competence partially supporting the idea that gamification can foster BPN satisfaction in PE. Although this global interaction did not derive from statistically significant post-hoc pairwise comparisons, descriptive trends favored both gamified interventions. Specifically, a descriptive tendency toward maintenance or slight improvement in perceived competence was observed within the ExMG and InMG, contrasting with the downward trajectory experienced by the CG. While the descriptive trends suggest that both gamified designs may have helped stabilize students’ perceived competence, neither profile demonstrated statistical superiority over the other in this specific psychological need. From a statistical standpoint, the presence of a significant omnibus interaction alongside non-significant post-hoc pairwise comparisons represents a well-documented outcome in factorial designs. While multiple statistical mechanisms concur, this pattern is primarily driven by the small-to-moderate magnitude of the effect ($\eta_{p}^{\text{2}}=.\text{0}\text{3}\text{6}$ for competence and $\eta_{p}^{\text{2}}=.\text{0}\text{3}\text{7}$ for future PA intention) and its distributed nature across all three experimental arms. Rather than a sharp, localized divergence between two specific conditions, the omnibus interaction reflects a system-wide structural divergence: the combined stabilizing/slight upward trajectories in both gamified groups operating in opposition to the progressive downward slope in the control arm. Because the explained variance is modest and spread across the three-group framework (resulting in post-intervention mean differences of approximately 0.1–0.2 points) no single pairwise contrast holds sufficient isolated signal. When these pair-by-pair contrasts are subsequently adjusted via Holm’s conservative correction, post-hoc statistical power is further attenuated, preventing any single comparison from reaching adjusted significance. Consequently, these interactions should be substantively interpreted as a collective protective buffering effect across the gamified arms against baseline psychological decline, rather than evidence of statistically decisive superiority between specific intervention profiles. Regarding the remaining BPNs, no interaction effect was found in the autonomy and relatedness variables. Nevertheless, there was no deterioration in these variables either.

Consequently, the findings only partially align with the initial hypotheses of the study and with what has been observed in gamification studies carried out in PE classes such as Quintas et al. [29] and Sotos-Martínez et al. [14]. On the one hand, Quintas et al. [29] applied an intervention programme based on gamification using the Just Dance Now exergame, stating improvements in BPN. Similarly, Sotos-Martínez et al. [14] conducted an intervention using the ClassCraft® gamified ICTtool. In this case, the results showed an improvement in the three BPN dimensions (autonomy, competence and relatedness). In contrast to these multi-dimensional improvements, our findings expose a more constrained impact, establishing a significant interaction exclusively driven by the trends in perceived competence.

Furthermore, the descriptive decline observed in the control group regarding perceived competence and future PA intentions could be associated with the gradual psychological challenges often observed in traditional secondary school PE settings [1–5]. In these conventional environments, the lack of innovative pedagogical tracking or narrative scaffolding may sometimes link to a progressive erosion of students’ psychological need satisfaction and behavioral intentions over time [4,5]. Within this framework, we speculatively propose, based on a post-hoc observation of the descriptive data, that the gamified interventions might have operated as a protective buffer, stabilizing students’ BPN and future intentions, and preventing the baseline decline observed in the control condition. Therefore, these descriptive trends suggest that deploying any structured gamification framework might offer a potential alternative to non-gamified traditional settings for preserving perceived competence. However, this interpretation must remain highly cautious and tentative, as it relies on non-significant within-group declines in the control condition and lacks significant pairwise comparisons.

Nonetheless, the present study only revealed a significant interaction and descriptive upward trend for perceived competence, without significant changes in autonomy and relatedness. These divergent results among the three BPNs could be associated with the distinct mechanisms through which specific game elements interface with psychological needs [10]. As noted by Peng et al. [22], the inclusion of explicit game features designed to track performance and display achievements can effectively support students’ need satisfaction of competence. However, our findings suggest that while these direct digital feedback mechanics can modulate competence satisfaction within a short timeframe, shifting autonomy and relatedness within an intact educational setting remains more challenging. Shifting students’ perception of autonomy requires a fundamental transfer of choice and control, while enhancing relatedness depends heavily on transforming the peer social climate [5]. In pre-existing secondary school class groups, social hierarchies, peer relationships, and traditional instructional habits are often firmly established [4]. Consequently, an 8-session intervention conducted over four weeks might be sufficient to support competence through concrete performance metrics, but may remain limited in altering the broader socio-ecological and interpersonal dynamics required to significantly shift autonomy and relatedness satisfaction.

It is also relevant to note a distinct interaction effect on the future PA intention variable, highlighted by a descriptive upward tendency uniquely within the InMG. This specific divergence provides a descriptive indication of a potential differential impact between the gamification profiles, suggesting that intrinsically oriented elements (such as narratives and cooperative missions) might have a promising role in translating short-term classroom experiences into intentions for future physical practice than extrinsic reward systems. However, this interpretation must be approached with caution. As with perceived competence, the significant omnibus interaction (p = .020, η²p = .037) captures the overall divergence between the upward trend in the InMG and the downward trajectory of the control group. However, because this effect is modest and individual variability is present across conditions, conservative Holm-adjusted pairwise comparisons did not reach statistical significance. Thus, while the global model confirms a differential temporal pattern across intervention arms, direct pairwise superiority cannot be conclusively claimed. This effect could be explained by Vasconcellos et al. [30], who, drawing on SDT, noted that numerous studies have shown that the greater the degree of intrinsic motivation is, the more favorable the results are in terms of extracurricular PA intention. This finding is also corroborated by Trigueros et al. [31], who explain that self-determined responses are a predictor of the formation of behaviors related to future PA practices.

Regarding motivational regulations, the statistical analyses revealed that neither the intrinsic nor the extrinsic gamification intervention induced significant shifts along the self-determination continuum. This lack of change across the different PLOC-R subscales could be associated with the conceptual distinction between situational interest and contextual motivation, alongside the specific nature of the physical tasks. Short-term research in PE often reports rapid motivational shifts when introducing completely alternative or novel physical activities, which temporarily reset peer competence hierarchies because all students start learning from a similar baseline [4,5]. By contrast, the present study evaluated contextual motivation [28] (reflecting students’ stable regulatory styles toward the subject as a whole) while keeping the physical curriculum and motor tasks identical across all conditions. Because the core physical experience involved traditional curricular contents and only the tracking framework was altered via *MyClassGame*, the intervention avoided artificial situational spikes driven by novel physical formats. According to the pathways identified by Rahman et al. [32], improvements in basic needs operate as precursors to motivation, but the actual internalization of these psychological gains into stable regulatory mechanisms occurs gradually over time. Consequently, an 8-session intervention appears sufficient to support the immediate cognitive appraisal of competence, but remains limited when attempting to alter deeply rooted contextual regulations within a compressed timeframe, regardless of whether the digital environment is informational or controlling.

In terms of contributions, this study addresses the lack of literature comparing the effects of different types of gamification elements on variables such as BPN and motivation [33]. By directly comparing two distinct gamification profiles within the same Physical Education context, this research moves beyond the excessive use of PBL or “pointification” [15,34]. Therefore, the study focuses on providing evidence regarding how these different structural configurations operate within this educational setting.

However, it is necessary to critically reflect on the dichotomous classification of these elements. While this study utilized theoretical frameworks to design “intrinsically” and “extrinsically” oriented profiles, game elements are not inherently fixed in their motivational impact. According to Cognitive Evaluation Theory [16], the psychological effect of any gamified element depends in part on whether the student perceives it as informational (thereby supporting competence) or controlling (thereby thwarting autonomy) [15,21]. Therefore, the InMG and ExMG categories reflect our pedagogical design intentions rather than fixed psychological boundaries, highlighting that the distinction between both forms of gamification is flexible in practice.

Beyond these theoretical nuances, the practical implementation of these pedagogical designs provides valuable insights into how different gamification profiles impact student outcomes. In this regard, while our data shows that both gamification types showed similar descriptive tendencies for supporting a slight increase in perceived competence compared to traditional methods, the descriptive patterns suggest a potential advantage for the profile utilizing elements designed to target intrinsic motivation when the goal is to promote future physical practice intentions. Nevertheless, despite the relevance and innovative aspects of the study, certain methodological constraints should be considered when interpreting the findings.

First, the time factor could have been a significant limitation. The 4-week intervention might not have been sufficient to achieve substantial changes in more motivation-related variables. In fact, most experimental studies on gamification have an average duration longer than 4 weeks [34,35]. Moreover, the quasi-experimental design utilizing pre-existing class groups introduces a potential selection bias and a nested data structure (students clustered within intact classes). To statistically account for this clustering within classes and safeguard internal validity, we initially updated our analytical framework to Linear Mixed Models (LMM), specifying class groups as a random factor. However, as detailed in the Statistical Analysis section, due to near-zero cluster variance and singular fits across most outcomes, the classroom-level random intercept was ultimately retained exclusively for Perceived Competence and Relatedness. For the remaining outcomes, model structures were simplified to participant-level random intercepts or, in the specific cases of Autonomy and External Regulation, adjusted via Repeated Measures ANCOVAs (RM-ANCOVA) due to persistent baseline collinearity. While these statistical adjustments were necessary to avoid overparameterization and ensure model convergence, the inability to retain a uniform full-multilevel hierarchy across all variables remains a methodological constraint of this field study. Furthermore, regarding the ecological validity of the gamification design, the interventions were fully embedded within a real-world school setting using identical traditional physical curricula. While this maximizes the ecological applicability of the findings to everyday PE contexts, it introduces complex socio-ecological variations that are difficult to isolate completely. Additionally, it must be explicitly acknowledged that all psychological and motivational findings in this study are based exclusively on self-report measures, which may be subject to social desirability or interpretation biases. Furthermore, a possible alternative explanation for the lack of significant effects on autonomy and relatedness is the absence of a subjective manipulation check. Without such a measure, we cannot verify if the students in the InMG actually perceived greater autonomy support than those in the ExMG, meaning the two conditions might not have been sufficiently differentiated in the students’ subjective experience despite their structural differences. Accordingly, caution is warranted when generalizing these results, and future research employing fully randomized cluster designs and incorporating objective behavioral measures is encouraged.

## Conclusions

In conclusion, the results illustrate the specific impact of each gamification profile evaluated in this study. Rather than demonstrating an absolute superiority of one design over another, the results indicate that both the intrinsically and extrinsically oriented gamification frameworks (InMG and ExMG) were associated with a descriptive tendency toward a slight improvement in students’ perceived competence when compared to our traditional non-gamified instruction group. However, these interpretations must be contextualized by the small effect sizes and the absence of significant post-hoc pairwise differences. Consequently, rather than indicating a definitive superiority, the InMG profile only showed a descriptive trend toward fostering intentions for future physical practice among our sample, while contextual motivational regulations remained stable and resistant to changes across all three experimental conditions.

## Supporting information

S1 File Classification of gamification elements across the InMG and ExMG interventions based on the Octalysis Framework and Self-Determination Theory. (DOCX)

S2 File Comprehensive statistical breakdown of post-hoc pairwise comparisons, estimated marginal means, and effect sizes across all conditions. (XLSX)

S3 File Dataset. (XLSX)

## References

[1] B Owen K, Smith J, Lubans DR, Ng JYY, Lonsdale C. Self-determined motivation and physical activity in children and adolescents: a systematic review and meta-analysis. Prev Med. 2014;67:270–9. doi: 10.1016/j.ypmed.2014.07.033 25073077

[2] Fernandez-Rio J, Sanz N, Fernandez-Cando J, Santos L. Impact of a sustained cooperative learning intervention on student motivation. Phy Edu Sport Pedagogy. 2016;22(1):89–105. doi: 10.1080/17408989.2015.1123238

[3] Martinac Dorčić T, Smojver-Ažić S, Božić I, Malkoč I. Effects of social media social comparisons and identity processes on body image satisfaction in late adolescence. Eur J Psychol. 2023;19(2):220–31. doi: 10.5964/ejop.9885 37731891 PMC10508212

[4] Hemingway K, Butt J, Spray C, Olusoga P, Beretta DeAzevedo L. Exploring students experiences of secondary school Physical Education in England. Phy Edu Sport Pedagogy. 2025;30: 576–91.doi: 10.1080/17408989.2023.2256771

[5] White RL, Bennie A, Vasconcellos D, Cinelli R, Hilland T, Owen KB, et al. Self-determination theory in physical education: a systematic review of qualitative studies. Teach Teacher Edu. 2021;99:103247. doi: 10.1016/j.tate.2020.103247

[6] Ryan RM, Deci EL. Intrinsic and extrinsic motivation from a self-determination theory perspective: definitions, theory, practices, and future directions. Contemp Edu Psychol. 2020;61:101860. doi: 10.1016/j.cedpsych.2020.101860

[7] Zourmpakis A-I, Kalogiannakis M, Papadakis S. Adaptive gamification in science education: an analysis of the impact of implementation and adapted game elements on students’ motivation. Computers. 2023;12(7):143. doi: 10.3390/computers12070143

[8] Werbach K, Hunter D. For the win: How game thinking can revolutionize your business. United States: Wharton Digital Press; 2012. http://wdp.wharton.upenn.edu/books/for-the-win/

[9] Tudela-Petit M, Marco-Ahulló A, García-Massó X. Revisión narrativa sobre la gamificación en Educación Física: desde la “puntificación” hasta la gamificación adaptativa: Narrative Review of Gamification in Physical Education: From “Pointification” to Adaptive Gamification. Papeles. 2024;16: 11–31. doi: 10.54104/papeles.v16n32.1975

[10] Deterding S. Situated motivational affordances of game elements: A conceptual model. CHI Gamification Workshop; 2011; 3–6.

[11] Gómez-Ejerique C, López-Cantos F. Application of innovative teaching-learning methodologies in the classroom. Coaching, flipped-classroom and gamification. A case study of success. MultJEduSocTecSci. 2019;6(1):46. doi: 10.4995/muse.2019.9959

[12] Arufe-Giráldez V, Sanmiguel-Rodríguez A, Ramos-Álvarez O, Navarro-Patón R. Gamification in physical education: a systematic review. Edu Sci. 2022;12(8):540. doi: 10.3390/educsci12080540

[13] Zhang Q, Yu Z. Meta-analysis on investigating and comparing the effects on learning achievement and motivation for gamification and game-based learning. Education Research International. 2022; 1519880. doi: 10.1155/2022/1519880

[14] Sotos-Martínez VJ, Ferriz-Valero A, García-Martínez S, Tortosa-Martínez J. The effects of gamification on the motivation and basic psychological needs of secondary school physical education students. Phy Edu Sport Pedagogy. 2022;29(2):160–76. doi: 10.1080/17408989.2022.2039611

[15] Huang R, Ritzhaupt AD, Sommer M, Zhu J, Stephen A, Valle N, et al. The impact of gamification in educational settings on student learning outcomes: a meta-analysis. Edu Tech Res Dev. 2020;68(4):1875–901. doi: 10.1007/s11423-020-09807-z

[16] Deci EL, Ryan RM. Cognitive evaluation theory. Intrinsic motivation and self-determination in human behavior. Springer US; 1985. 43–85. doi: 10.1007/978-1-4899-2271-7_3

[17] Deci EL, Koestner R, Ryan RM. A meta-analytic review of experiments examining the effects of extrinsic rewards on intrinsic motivation. Psychol Bull. 1999;125(6):627–68; discussion 692-700. doi: 10.1037/0033-2909.125.6.627 10589297

[18] Navarro-Mateos C, Mora-Gonzalez J, Pérez-López IJ. The “STAR WARS: the first jedi” program-effects of gamification on psychological well-being of college students. Games Health J. 2024;13(2):65–74. doi: 10.1089/g4h.2023.0059 37856161

[19] Xu J, Lio A, Dhaliwal H, Andrei S, Balakrishnan S, Nagani U, et al. Psychological interventions of virtual gamification within academic intrinsic motivation: a systematic review. J Affect Disord. 2021;293:444–65. doi: 10.1016/j.jad.2021.06.070 34252688

[20] Camacho-Sánchez R, Manzano-León A, Rodríguez-Ferrer JM, Serna J, Lavega-Burgués P. Game-based learning and gamification in physical education: a systematic review. Education Sciences. 2023;13(2):183. doi: 10.3390/educsci13020183

[21] Forde SF, Mekler ED, Opwis K. Informational vs. controlling gamification: a study design. proceedings of the 2015 annual symposium on computer-human interaction in play. New York, NY, USA: Association for Computing Machinery; 2015. 517–22. doi: 10.1145/2793107.2810297

[22] Peng W, Lin J-H, Pfeiffer KA, Winn B. Need satisfaction supportive game features as motivational determinants: an experimental study of a self-determination theory guided exergame. Media Psychology. 2012;15(2):175–96. doi: 10.1080/15213269.2012.673850

[23] Chou Y. Actionable gamification: beyond points, badges, and leaderboards. Packt Publishing Ltd; 2019.

[24] Verjans-Janssen SRB, Van Kann DHH, Gerards SMPL, Vos SB, Jansen MWJ, Kremers SPJ. Study protocol of the quasi-experimental evaluation of “KEIGAAF”: a context-based physical activity and nutrition intervention for primary school children. BMC Public Health. 2018;18(1):842. doi: 10.1186/s12889-018-5764-3 29980235 PMC6035437

[25] Sebire SJ, Jago R, Fox KR, Edwards MJ, Thompson JL. Testing a self-determination theory model of children’s physical activity motivation: a cross-sectional study. Int J Behav Nutr Phys Act. 2013;10:111. doi: 10.1186/1479-5868-10-111 24067078 PMC3852537

[26] Chatzisarantis NLD, Biddle SJH, Meek GA. A self‐determination theory approach to the study of intentions and the intention–behaviour relationship in children’s physical activity. British J Health Psychol. 1997;2(4):343–60. doi: 10.1111/j.2044-8287.1997.tb00548.x

[27] Castillo I, Molina-García J, Estevan I, Queralt A, Álvarez O. Transformational teaching in physical education and students’ leisure-time physical activity: the mediating role of learning climate, passion and self-determined motivation. Int J Environ Res Public Health. 2020;17(13):4844. doi: 10.3390/ijerph17134844 32635673 PMC7370029

[28] Moreno Murcia JA, González-Cutre Coll D, Chillón Garzón M. Preliminary validation in Spanish of a scale designed to measure motivation in physical education classes: the Perceived Locus of Causality (PLOC) scale. Span J Psychol. 2009;12(1):327–37. doi: 10.1017/s1138741600001724 19476244

[29] Quintas A, Bustamante J-C, Pradas F, Castellar C. Psychological effects of gamified didactics with exergames in Physical Education at primary schools: Results from a natural experiment. Comp Edu. 2020;152:103874. doi: 10.1016/j.compedu.2020.103874

[30] Vasconcellos D, Parker PD, Hilland T, Cinelli R, Owen KB, Kapsal N, et al. Self-determination theory applied to physical education: a systematic review and meta-analysis. J Edu Psychol. 2020;112(7):1444–69. doi: 10.1037/edu0000420

[31] Trigueros R, Aguilar-Parra JM, Cangas AJ, Fernández-Batanero JM, Álvarez JF. The influence of the social context on motivation towards the practice of physical activity and the intention to be physically active. Int J Environ Res Public Health. 2019;16(21):4212. doi: 10.3390/ijerph16214212 31671662 PMC6862659

[32] Rahman RJ, Thogersen-Ntoumani C, Thatcher J, Doust J. Changes in need satisfaction and motivation orientation as predictors of psychological and behavioural outcomes in exercise referral. Psychol Health. 2011;26(11):1521–39. doi: 10.1080/08870446.2010.538849 22111661

[33] Elderen J, Stappen E. The potential impact of gamification elements on the acceptance of technology in the context of education: a literature review. BLED 2019 Proceedings. 2019. https://aisel.aisnet.org/bled2019/51

[34] Manzano-León A, Camacho-Lazarraga P, Guerrero MA, Guerrero-Puerta L, Aguilar-Parra JM, Trigueros R, et al. Between level up and game over: a systematic literature review of gamification in education. Sustainability. 2021;13(4):2247. doi: 10.3390/su13042247

[35] Mula-Falcon J, Moya-Roselo I, Ruiz-Ariza A. The active methodology of gamification to improve motivation and academic performance in educational context: a meta-analysis. Rev Eur Stud. 2022;14: 32–46. 10.5539/res.v14n2p32

