# Supplementary material for: Exploring the impact of gamified elements on motivation-related variables in physical education
Source: PLoS One. 2026 Sep 23;21(9):e0358950. doi: 10.1371/journal.pone.0358950 (PMC13600532; doi:10.1371/journal.pone.0358950)
Supplement: Supplementary file 1 [file pone.0358950.s001.docx]

**Table S1. Description of the game elements used in the study.**

| **Group** | **Game Element** | **Octalysis Core Drive** | **Description** | **BPN targered** | **Informational vs Controlling function** |
| --- | --- | --- | --- | --- | --- |
| ExMG (Extrinsic) | Status Points (XP) | Accomplishment (#2) | Points accumulated for visible achievements and quantifiable progress. | CT, AT | Mostly informational, but can become controlling if tied to comparison |
|  | Badges | Accomplishment (#2) | Badges awarded for specific milestones, reinforcing achievements. | CT | Controlling (used as external reward) |
|  | Fixed Action Rewards | Accomplishment (#2) | Rewards obtained after completing predefined actions. | CT | Controlling |
|  | Leaderboard | Accomplishment (#2) | Public ranking based on score, promotes comparison and competition. | CT, AT | Controlling |
|  | Progress Bar | Accomplishment (#2) | Visual indicator of progress in the gamified experience. | CS | Informational |
|  | Desert Oasis | Accomplishment (#2) | A rare moment of abundance or reward placed in an otherwise empty or unrewarding environment, making the experience more memorable and valued. | CT, AT | Controlling |
|  | Boss Fights | Accomplishment (#2) | Challenging tasks that mark progression and offer valuable rewards. | CT | Controlling if challenge is not attainable |
|  | Milestone unlocks | Empowerment (#3) | Unlocking new content after cooperative goals are met. | CS | Informational |
|  | Boosters | Empowerment (#3) | Items that temporarily enhance avatar performance. | CS, AS | Informational |
|  | Exchangeable Points | Ownership (#4) | Virtual currency is used to redeem items or advantages. | CS | Controlling |
|  | Virtual Goods | Ownership (#4) | Digital items reinforcing the sense of possession. | AT | Controlling |
|  | Build from Scratch | Ownership (#4) | Allows users to create or develop something from the ground up, fostering ownership, investment, and creativity. | AS | Informational |
|  | Collection Sets | Ownership (#4) | Sets of items to be collected, fostering completion desire. | CT | Mixed to controlling, can become compulsive |
|  | Avatar | Ownership (#4) | Customizable graphic representation of the user. | AS | Informational |
|  | Protector Quest | Ownership (#4) | Missions where the user’s role is to protect or help others, fostering responsibility, empathy, and social connection. | RS | Informational |
|  | Friending | Social Influence (#5) | Social relationships in the game for collaboration or helping others. | RS | Informational |
|  | Appointment Dynamics | Scarcity (#6) | Rewards available only at certain times, generating urgency. | AT | Controlling |
|  | Magnetic Caps | Scarcity (#6) | Hard-to-get items that increase perceived value. | CT, AT | Controlling |
|  | Dangling | Scarcity (#6) | A reward or objective is shown to the user but kept just out of reach, triggering curiosity and anticipation. | AT | Controlling |
|  | Prize Pacing | Scarcity (#6) | Staggered reward distribution to maintain interest. | CT | Controlling |
|  | Bootleg Quest | Scarcity (#6) | Unofficial or hidden missions that are not part of the main storyline, encouraging exploration, creativity, and a sense of rebellion. | AS | Informational |
|  | Last Mile Drive | Scarcity (#6) | End-of-task incentives to prevent dropout. | CT | Controlling through pressure |
|  | Moats | Scarcity (#6) | Barriers or exclusive areas that require effort, status, or resources to access, increasing perceived value and prestige. | AT, RT | Controlling |
|  | Big Burn | Scarcity (#6) | Optional or difficult quests that add reputation or punishment if failed. | CT | Controlling via frustration |
|  | Progress Loss | Avoidance (#8) | Loss of previously earned progress when the user is inactive or underperforms. | CT, AT | Controlling |
|  | Rightful Heritage | Avoidance (#8) | A reward or status the user feels pressured to reclaim in order to maintain what is perceived as already theirs. | AT | Controlling |
|  | Evanescence Opportunity | Avoidance (#8) | A limited-time reward or event that disappears if not used promptly, creating urgency and FOMO (Fear of missing out). | AT, CT | Controlling |
|  | Streaking | Avoidance (#8) | Maintaining a consecutive sequence of actions (e.g., daily tasks) in a way that may make setbacks more discouraging. | CT | Controlling |
| InMG (Intrinsic) | Narrative | Epic Meaning (#1) | Central story that provides meaning and purpose to the experience. | AS, RS | Informational |
|  | Elitism | Epic Meaning (#1) | Motivation derived from belonging to an exclusive group perceived as special or prestigious. | RS | Informational; can support belonging |
|  | Humanity Hero | Epic Meaning (#1) | Students’ symbolic role as world saviors. | AS, RS | Informational |
|  | Revealed Heart | Epic Meaning (#1) | Narrative moments that deepen emotional connection with the story. | RS | Informational |
|  | Beginner’s Luck | Epic Meaning (#1) | Initial advantage that builds confidence and curiosity. | CS | Informational |
|  | Free Lunch | Epic Meaning (#1) | Unexpected rewards that encourage exploration and engagement. | CS | Informational |
|  | Cap Switcher | Epic Meaning (#1) | Tool for modifying roles or abilities based on player decisions. | AS | Informational |
|  | Badges | Accomplishment (#2) | Badges awarded for specific milestones, reinforcing achievements and mastery-linked. | CS | Informational |
|  | Boss Fights | Accomplishment (#2) | Same as ExMG, but embedded in narrative, mastery, and cooperation. | CS | Informational |
|  | Milestone Unlocks | Empowerment (#3) | Unlocking new content after cooperative goals are met. | CS | Informational |
|  | Real-Time Control | Empowerment (#3) | Enables users to make immediate decisions that directly influence outcomes, enhancing autonomy and engagement. | AS | Informational |
|  | Chain Combos | Empowerment (#3) | A sequence of actions that trigger escalating rewards when performed in rapid succession, reinforcing competence and flow. | CS | Informational |
|  | Dynamic Feedback | Empowerment (#3) | Instant, adaptive responses to user actions that guide progress and reinforce performance in real time. | AS, CS | Informational |
|  | Boosters | Empowerment (#3) | Items that temporarily enhance avatar performance and were player-chosen | AS, CS | Informational |
|  | Plant Pickers & Poison Pickers | Empowerment (#3) | Choices that appear equal but have hidden positive or negative outcomes, encouraging strategic thinking and risk assessment. | AS, CS | Informational |
|  | Attribute Web Chart | Empowerment (#3) | A visual map showing how a player’s attributes evolve based on their decisions, reinforcing self-reflection and personalized progression. | CS | Informational |
|  | Double-Edged Sword | Empowerment (#3) | Game elements that provide both advantages and disadvantages, requiring the user to make meaningful trade-offs. | AS | Informational |
|  | Avatar | Ownership (#4) | Customizable graphic representation of the user. | AS | Informational |
|  | Friending | Social Influence (#5) | Social relationships in the game for collaboration or helping others. | RS | Informational |
|  | Social Treasure | Social Influence (#5) | Rewards or benefits that can only be unlocked through social interaction or cooperation with others. | RS | Informational |
|  | Group Quests | Social Influence (#5) | Missions that require collaboration among multiple users to succeed, promoting teamwork and shared goals. | CS, RS | Informational |
|  | Trophy Self | Social Influence (#5) | A visual or symbolic representation of the user’s status or achievements, designed to impress others. | CS, RS | Informational |
|  | Mentorship | Social Influence (#5) | A mechanic that allows experienced users to support newcomers, fostering connection, guidance, and responsibility. | RS | Informational |
|  | Social Proud | Social Influence (#5) | A sense of pride and recognition gained from being admired or respected within a social group or community. | RS | Informational |
|  | Moats | Scarcity (#6) | Unlockable barriers or areas that require effort and successful progression to access, reinforcing competence through earned advancement | CS | Informational |
|  | Glowing Choice | Unpredictability (#7) | Highlighted options that subtly guide user decisions while maintaining a sense of autonomy and discovery. | AS | Informational |
|  | Mini Quests | Unpredictability (#7) | Small, optional challenges embedded in the experience to encourage exploration, mastery, or progression. | AS, CS | Informational |
|  | Visual Story Telling | Unpredictability (#7) | Use of compelling images or animations to convey narrative, evoke emotion, and enhance engagement. | AS, RS | Informational |
|  | Easter Egg | Unpredictability (#7) | Hidden messages, features, or rewards which users can discover through curiosity or experimentation. | AS, CS | Informational |
|  | Random Rewards | Unpredictability (#7) | Prizes or outcomes that appear unpredictably, increasing excitement and anticipation. | CS | Informational |
|  | Obvious Wonder | Unpredictability (#7) | Clearly visible but unexplained elements designed to spark curiosity and encourage users to explore further. | AS | Informational |
|  | Rolling Rewards | Unpredictability (#7) | Systems that increase the quantity or quality of rewards the longer a user stays engaged. | CS | Informational |
|  | Sudden Rewards | Unpredictability (#7) | Unexpected positive reinforcements that appear without warning, surprising and delighting the user. | CS | Informational |
|  | Oracle Effect | Unpredictability (#7) | When the system seems to anticipate or respond meaningfully to user behavior, creating a sense of intelligence and personalization. | AS, CS | Informational |
|  | Progress Loss | Avoidance (#8) | Loss of previously earned progress when the user is inactive or underperforms, encouraging consistent engagement. | AS, CS | Informational |
|  | Rightful Heritage | Avoidance (#8) | A reward or status the user feels entitled to but must reclaim, creating emotional drive and perceived ownership | AS | Informational |
|  | Evanescence Opportunity | Avoidance (#8) | A limited-time opportunity designed to encouraged timely engagement with specific activities or rewards. | CS | Informational |
|  | Streaking | Avoidance (#8) | Maintaining a consecutive sequence of actions (e.g., daily tasks) to build habit and avoid breaking the chain. | CS | Informational |
|  | Devil Egg | Avoidance (#8) | A hidden, often risky feature that may yield a reward or punishment, enhancing tension. | CS | Informational |
|  | FOMO punch | Avoidance (#8) | A sharp emotional reminder that others are benefiting from something you’re missing out on. | AS | Informational |

Note. AT= autonomy-thwarting; CT= competence-thwarting; RT= relatedness-thwarting; AS= autonomy-supportive; CS= competence-supportive; RS= relatedness-supportive.
